# Comprehensive analysis of protein acetyltransferases of human pathogen *Mycobacterium tuberculosis*

**DOI:** 10.1042/BSR20191661

**Published:** 2019-12-20

**Authors:** Longxiang Xie, Wenmin Yang, Xiangyu Fan, Jianping Xie

**Affiliations:** 1 Institute of Modern Biopharmaceuticals, State Key Laboratory Breeding Base of Eco-Environment and Bio-Resource of the Three Gorges Area, Key Laboratory of Eco-environments in Three Gorges Reservoir Region, Ministry of Education, School of Life Sciences, Southwest University, Beibei, Chongqing 400715, China; 2 Institute of Biomedical Informatics, Cell Signal Transduction Laboratory, Bioinformatics Center, School of Basic Medical Sciences, Henan University, Kaifeng, Henan 475004, China; 3 School of Biological Science and Technology, University of Jinan, Shandong 250022, China

**Keywords:** acetylation, acetyltransferase, mycobacterium, Tuberculosis, virulence

## Abstract

Tuberculosis (TB), a leading infectious disease caused by *Mycobacterium tuberculosis* strain, takes four human lives every minute globally. Paucity of knowledge on *M. tuberculosis* virulence and antibiotic resistance is the major challenge for tuberculosis control. We have identified 47 acetyltransferases in the *M. tuberculosis*, which use diverse substrates including antibiotic, amino acids, and other chemical molecules. Through comparative analysis of the protein file of the virulent *M. tuberculosis* H37Rv strain and the avirulent *M. tuberculosis* H37Ra strain, we identified one acetyltransferase that shows significant variations with N-terminal deletion, possibly influencing its physicochemical properties. We also found that one acetyltransferase has three types of post-translation modifications (lysine acetylation, succinylation, and glutarylation). The genome context analysis showed that many acetyltransferases with their neighboring genes belong to one operon. By data mining from published transcriptional profiles of *M. tuberculosis* exposed to diverse treatments, we revealed that several acetyltransferases may be functional during *M. tuberculosis* infection. Insights obtained from the present study can potentially provide clues for developing novel TB therapeutic interventions.

## Introduction

TB remains a major threat for global health largely due to vast mortality and morbidity. In 2017, an estimated 17 million people were infected by TB and 1.6 million died from this disease (WHO, 2018). The increase of incidences with MDR_TB (multidrug resistant TB) and XDR_TB (extensively drug-resistant TB) further exacerbates the difficulty of TB patients’ treatment. It is imperative to find new targets and new anti-TB drugs. *M. tuberculosis* is the causative agent of TB and has developed the ability to survive in a dormant state for long time under stress conditions within its human host or to resist various antibiotics [1]. Rv0262c, a conserved aminoglycoside 2′-N-acetyltransferase (AAC(2′)-Ic) in mycobacteria, can acetylate all known aminoglycosides including ribostamycin, neomycin B, gentamicin, and tobramycin bearing 2′ amino group [2,3]. Moreover, Rv0262c is capable of performing O-acetylation in kanamycin A and amikacin at 2′ position [4]. Rv3225c, a putative phophotransferase containing GNAT (GCN5-related acetyltransferase) domain in N terminus, was found to have low level of aminoglycoside-modifying activity conferring resistance to aminoglycoside antibiotic in mycobacteria [5]. *Eis* (*rv2416c*, **e**nhanced **i**ntracellular **s**urvival gene), one lysine N-acetyltransferase, was found its overexpression conferring *M. tuberculosis* kanamycin resistance [6]. About 80% of clinical isolates harboring *eis* promoter mutations exhibited low-level kanamycin resistance [7]. In addition, Eis has an unexpected function of acetylating capreomycin thereby deactivating the drug [8]. In 2012, Green et al. screened three small molecule libraries and found 25 inhibitors that display specific and strong inhibitory activity against Eis *in vitro* [9]. Thus, these acetyltransferases has emerged as targets for inhibitor design.

Integrity of cell envelope is crucial for *M. tuberculosis* survival, virulence, and persistence [10]. PG (Peptidoglycan), mycolic acids, and AG (arabinogalactan) are the major constituents of cell envelope in *M. tuberculosis* [11]. GlcNAc (Amino sugar N-acetylglucosamine), a critical component of PG and UDP-GlcNAc (an activated type of amino sugar), is an indispensable precursor for different cell wall components [11]. Bi-functional enzyme GlmU (N-acetylglucosamine-1-phosphate uridyltransferase), encoded by *rv1018c* gene, contains both acetyltransferase and uridyltransferase domains [12,13]. GlmU is involved in the final two steps of UDP-N-acetyl-d-glucosamine (UDP-GlcN) biosynthesis process [12,13]. The deletion of *glmU* gene can change the cell wall structure, and GlmU is necessary for mycobacterial survival in THP-1 cells and guinea pigs [14]. In addition, GlmU interacting with IL-8 can facilitate the pathogen *M. tuberculosis* entry into human neutrophils [15]. In this work, we found the presence of acetyltransferases with different substrates by analyzing the *M. tuberculosis* proteome. Comparative proteomic analyses showed that there are homologues of several *M. tuberculosis* acetyltransferases in opportunistic and non-pathogenic mycobacteria. We also found that one acetyltransferase can be lysine acetylated, succinylated and glutarylated, and many acetyltransferases with their neighboring genes are conserved in mycobacteria.

## Materials and methods

### Identification of acetyltransferases in the *M. tuberculosis* proteome

All characterized and predicted acetyltransferases in the *M. tuberculosis* H37Rv proteome were identified by searching the keywords: ‘acetyltransferase’ and ‘acetylase’ in the NCBI (National Center for Biotechnology Information). The detailed workflow was shown in Supplementary Figure S1. The proteomes of the 14 mycobacterial species downloaded from NCBI ftp were used in this study (Supplementary Table S3). The genomic map was created by using the DNAplotter tool [16].

### Cross comparison of acetyltransferases in mycobacteria

*M. tuberculosis* H37Rv acetyltransferases found in the above step were compared with the other 13 species as shown in Supplementary Table S4 for the identification of homologous protein using Blast. Two proteins were treated as homologous: identity value ≥ 50%, query coverage ≥ 70%.

### Antigenic index, globularity, and physicochemical analyses

The following bioinformatics analysis followed previous report [17]. The Antigenic Index for all acetyltransferases was predicted through the VaxiJen v2 webserver, and the cut-off value was set 0.4 [18]. GlobPlot (http://globplot.embl.de/) website was conducted for searching the globularity and disorder in the acetyltransferases protein sequences [19]. The ProtParam tool of ExPASy (http://web.expasy.ogr/protparam/) was performed to predict the GRAVY (Grand Average of Hydropathicity), aliphatic indices, instability indices, and *in vivo* half-life of these proteins [20,21].

### Genomic context analysis

Acetyltransferases and their neighboring genes were screened to analyze their co-occurrence and predict functional associations through TB database (http://www.tbdb.org/) [22].

### Analysis of “omics information” related to acetyltransferases’ expression patterns and PTMs

All public transcriptomic and proteomic studies data were downloaded from the internet, and these data were used to retrieve the expression patterns of acetyltransferases under different conditions including oxygen-depleted model, nutrient starvation model, phagosome model, acid-nitrosative multi-stress, and mice model [23–27]. At least a 2-fold decrease or increase in the expression intensity of acetyltransferases is treated as significant. All post-translation modifications (PTMs) data including acetylation, succinylation, and glutarylation about acetyltransferases were obtained from our published articles [28–30].

## Results and discussion

### Classification of acetyltransferases in *M. tuberculosis*

**Figure 1:**
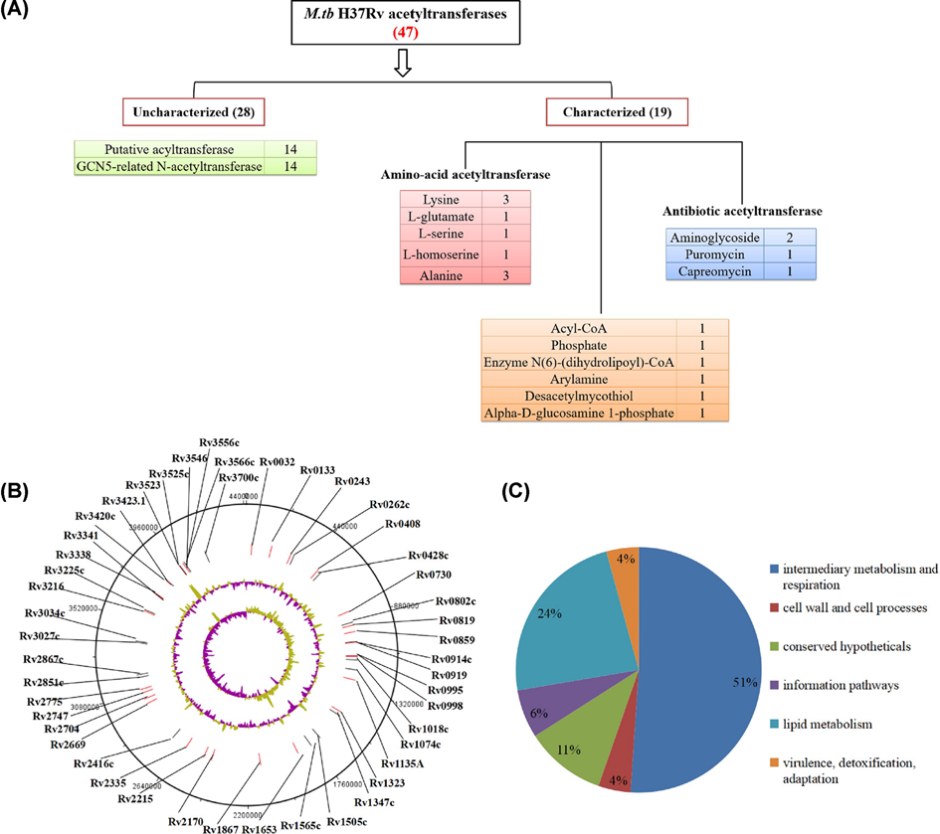
Summary of 47 potential acetyltransferases in *M.tuberculosis* (**A**) Functional classification of acetyltransferases found in *M. tuberculosis* H37Rv. All the acetyltransferases were classified according to their different substrate specificites. (**B**) Genomic map showing the coordinates of the 47 acetyltransferases identified in *M. tuberculosis* H37Rv. Acetyltransferases constitute 1.2% of the *M. tuberculosis* proteome. The tracks from the outside represent: (1) 47 genes’ positions in the genome, (2) %GC plot, and (3) GC skew (GC)/(G+C). (**C**) Functional categorization of *M. tuberculosis* H37Rv acetyltransferases.

All acetyltransferases including characterized, possible, probable, or hypothetical proteins were searched in the proteome of *M. tuberculosis* H37Rv, and a total of 47 acetyltransferases was found (Supplementary Table S1). Among these acetyltransferases, 9 are amino-acid acetyltransferase, 4 are antibotic acetyltransferases, 6 are other acetyltransferases, and the others are putative proteins without known functions. These proteins are mapped on a circle map with their Rv numbers (Figure 1A), representing 1.2% of the proteome of *M. tuberculosis*. Functional catagories of all the 47 acetyltransferases were retrieved from TubercuList database [31], and these proteins were divided into six categories based on their functionality as shown in Figure 1B. Twenty-four acetyltransferases belong to the intermediary metabolism and respiration category while eleven acetyltransferases belong to lipid metabolism category. These results not only show the variability of acetyltransferases, but also point to their probable functional importance.

### Cross proteomic comparison of *M. tuberculosis* acetyltransferases

**Figure 2:**
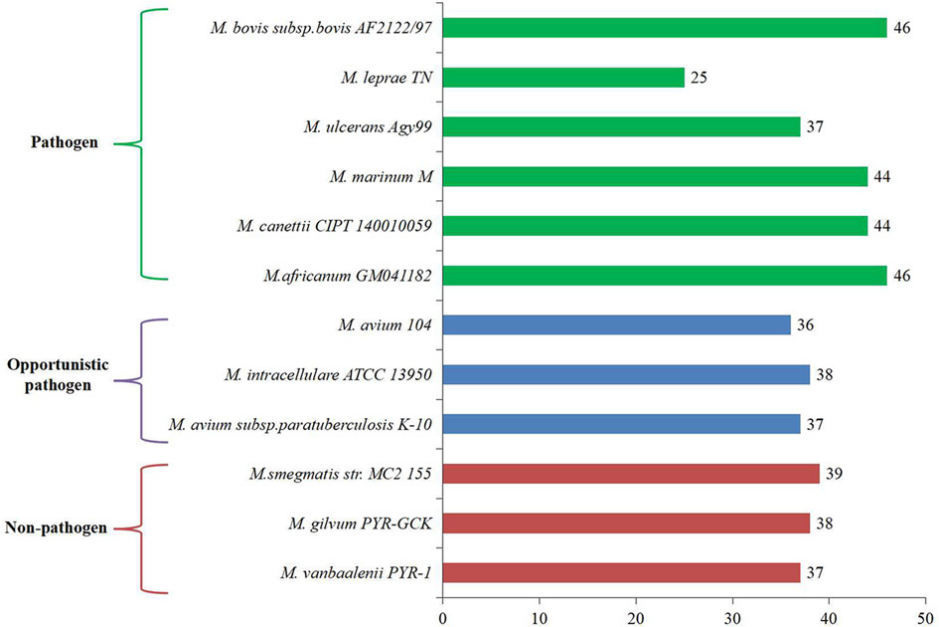
The homologues of 47 acetyltransferases in mycobacteria Comparison of 47 acetyltransferases between *M. tuberculosis* H37Rv with other mycobacterium species.

To explore whether these acetyltransferase and their homologues play an important role in the pathogenicity and metabolic processes of mycobacterium, these 47 acetyltransferases of *M. tuberculosis* were compared with various representative pathogenic, opportunistic and non-pathogenic mycobacteria species (a total 12) (Figure 2). *M. bovis* was found to have the highest number of homologous acetyltransferases in *M. tuberculosis*, while *M. leprae* had the lowest number of homologues. When moving from pathogenic to opportunistic to non-pathogenic bacteria, we found a reduction in the number of *M. tuberculosis* H37Rv homologues. On average strict pathogens have around 40 acetyltransferases; however, opportunistic and non-pathogenic mycobacteria have 37 and 38, respectively. Notably, the reduced number may be due to not synthesized in the species or the technical limitations resulting in unidentified acetyltransferases. Hence, the existence of these acetyltransferases in different mycobacterial species should be validated by experimental approaches.

### One potential acetyltransferase only present in the MTB complex

MTB complex including *M. tuberculosis, M. bovis, M. canetti, M. africanum, M. microti, M. pinnipedii, M. mungi, M. caprae*, and *M. suricattae* can cause tuberculosis in humans or other organisms [32]. Among the 47 putative acetyltransferases, Rv0032 (BioF2) annotated as putative 8-amino-7-oxononanoate synthase 2 with N-Acyltransferase superfamily region was only present in the MTB complex, but absent in other pathogenic, opportunistic pathogenic or non-pathogenic species of mycobacterium (Supplementary Table S2). Biotin, a critical micronutrient, is an essential enzyme cofactor for biotin-dependent enzymes such as pyruvate carboxylase and acyl-CoA carboxylase in *M. tuberculosis* [33]. Two BioF proteins are present in the *M. tuberculosis* genome, namely, BioF1 encoded by *rv1569* gene and BioF2 [34]. BioF1 is KAPA (7-Keto-8-aminopelargonic acid) synthase and is responsible for the first reaction step in the biotin biosynthesis pathway [33]. Using genome-wide genetic screens method, Sassetti et al. had found that *M. tuberculosis bioF* mutants (A and B) resulted in rapid clearance in the early phase of infection, and showed significantly decreased growth rates in mouse lung and spleen [35]. The growth of transposon insertion inactivated BioF1 *M. tuberculosis* was slowed, but returned to normal when supplemented with biotin [35]. However, the role of bioF2 in *M. tuberculosis* biotin metabolism remains unclear and the potential characteristics of acetyltransferase need to be verified by the experimental results. Notably, though BioF2 was only present in MTB complex, it does not mean that it is a specific enzyme to MTB just based on the current information. Furthermore, the other acetyltransferases that are shared between MTB complex and other mycobacterial species might also have essential functions in MTB complex, which need to be verified in the further study.

### Antigenicity profiling of *M. tuberculosis* acetyltransferases

We used a VaxiJen tool to analyze antigenicity of 47 acetyltransferases, and found 40 possible antigens and 7 non-antigenic proteins, which indicated that most of acetyltransferases proteins (85%) in *M. tuberculosis* are antigenic in nature, reflecting the immunomodulatory nature of these acetyltransferases. High antigenic regions within *M. tuberculosis* proteins have been reported involved in both humoral and T-cell responses *in vitro* or in clinical samples [36]. Mycolic acids, one important component of the unique cell wall in mycobacteria, constitute a bracket for lipid antigens to stimulate CD1-restricted T cells [37]. In 2004, Bhakta et al. demonstrated that Rv3566c (arylamine N-acetyltransferase, nat) is involved in the biosynthetic pathway of mycolic acids and complex lipids [38]. *nat* deletion *M. bovis* BCG strain showed multiple phenotypes such as postponed entry into log phase, changed morphology and lipid composition of cell wall, and increased intracellular killing rate by mouse macrophage cell line RAW264.7 [38].

### Comparison of acetyltransferases between *M. tuberculosis* H37Rv and H37Ra

**Figure 3:**
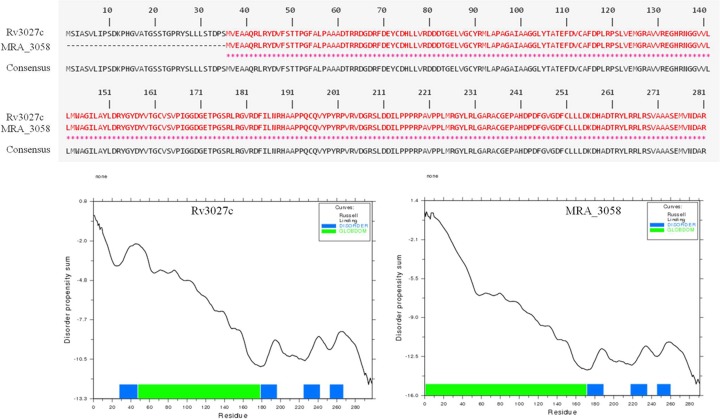
The difference of acetyltransferases between *M.tuberculosis* H37Rv and H37Ra Globular domain of Rv3027c and MRA_3058 predicted by GlobPlot. Amino acid sequence alignment indicates the difference between Rv3027c and MRA_3058.

Due to few virulence genes identified in *M. tuberculosis* [39]; therefore, it is urgent to find and identify the novel virulence genes. Virulent *M. tuberculosis* H37Rv strain is the main pathogen that can cause tuberculosis, while H37Ra strain loses the pathogenicity to a large extent [40]. The avirulent *M. tuberculosis* H37Ra strain is different from virulent *M. tuberculosis* H37Rv strain in many aspects including genomic insertion, deletion, and frame shift [40]. These two strains are the good material to find the potential virulence gene in *M. tuberculosis*. We analyzed protein sequence differences of acetyltransferases between H37Rv and H37Ra strain, and found that two acetyltransferases including Rv3027c and Rv3423.1 have a partial or whole deletion in the sequence length (Figure 3). *rv3027c* gene codes for possible GCN5-related N-acetyltransferase while its homologue in H37Ra, MRA_3058, has an N-terminal deletion (36aa), resulting in a 19.8% decrease in the hydrophilicity of the protein. Globular domains for proteins have special functions, whose deletion or addition might cause function loss or gain [41]. Comparing with Rv3027c, MRA_3058 has almost 45 amino acids increase in globular domain (Figure 3). Rv3423.1, one hypothetical protein, has no homologue in H37Ra. Recently, Joes et al. demonstrated that Rv3423.1 is a new histone acetyltransferase, which can acetylate histone H3 of host cell at the lysine 9/lysine 14 positions [42]. In addition, Rv3423.1 protein can be detected in the culture filtrate of virulent *M. tuberculosis* but not avirulent strains [42]. This indicates that these two acetyltransferases may play a critical role in the virulence of *M. tuberculosis* H37Rv and might underlie the attenuation of *M. tuberculosis* H37Ra.

### Comparison of acetyltransferases of *M. tuberculosis* versus *M. leprae*

*M. leprae*, the causative agent of leprosy, has much reduced genome than *M. tuberculosis* [43]. Both *M. tuberculosis* and *M. leprae* belong to prototypical intracellular pathogens that have evolved ways to survive in the intracellular phagosomes [44]. To explore whether these 47 acetyltransferases involve in the same cellular process, we compared the quantity difference of acetyltransferases between *M. tuberculosis* and *M. leprae*. Among the 47 acetyltransferases present in *M. tuberculosis*, 25 acetyltransferases have homologs while 22 proteins did not show any homology in *M. leprae*. We identified eight acetyltransferases, namely Rv1074c, Rv1135A, Rv1867, Rv3523, Rv3556c, Rv3566c, Rv2669 and Rv3027c, which are present in all other mycobacteria but absent in *M. leprae*. Rv1074c was identified from *M. tuberculosis* culture filtrate proteins [45]. Rv1867 was identified as potential tuberculosis drug target by comparative analyses of genomes from *M. tuberculosis* and human [46]. Rv3523 was predicted as cholesterol metabolism related protein [47]. Rv3556c is found to be expressed in S7 clinical strain but inhibited in laboratory H37Rv when bacteria encounter hypoxia condition [48]. Recent study had demonstrated that *kstR2* (a TetR-type transcriptional repressor) and *rv3556c* are de-repressed by cholesterol in *M. Tuberculosis* [49], implying the role in the control of cholesterol utilization. Rv1135Awith the condensing enzyme activity can catalyze a decarboxylating or non-decarboxylating Claisen-like condensation reaction and may involve in the synthesis and degradation of fatty acids [50]**.** *rv2669* gene was found to be up-regulated in *senX3–regX3* (two-component regulatory system) *M. tuberculosis* mutant strain [51]. This mutant strain showed the growth defect in the macrophages, and was attenuated in both immunocompetent and immunodeficient mice [51]. *M. tuberculosis* mutants in Rv3027c were attenuated for growth in macrophage [52].

### Overlap members of acetyltransferases with virulence factors and essential genes

**Figure 4:**
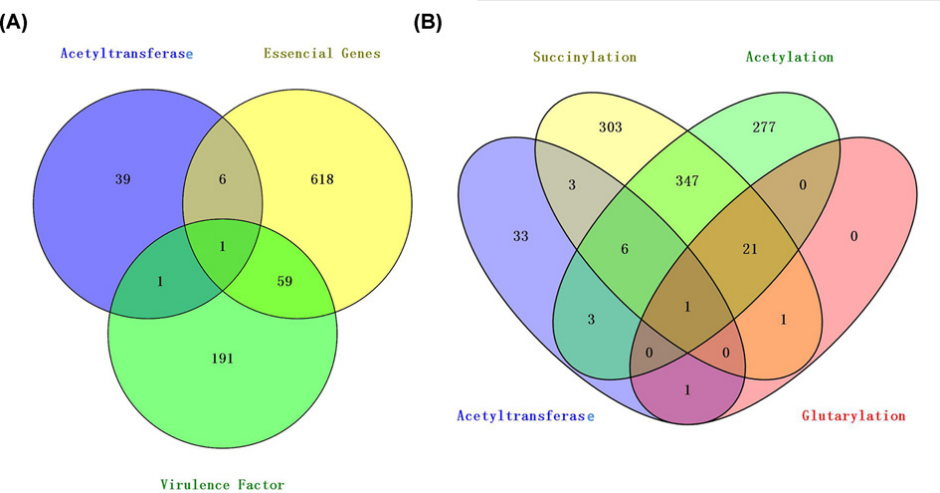
The characteristics of 47 acetyltransferases in virulence and PTMs (**A**) Comparison of *M. tuberculosis* H37Rv acetyltransferases with virulence factors and essential genes found in *M. tuberculosis* H37Rv. (**B**) Comparison of 47 acetyltransferases with three PTMs found in *M. tuberculosis* H37Rv.

Virulence factor database (VFDB) is a source for providing the current knowledge about virulence factors from different bacterial pathogens (http://www.mgc.ac.cn/VFs/) [53]. To understand whether these different acetyltransferases are related with virulence in *M. tuberculosis*, VFDB was searched, resulting two acetyltransferases of *M. tuberculosis*, Rv1347c (lysine N-acyltransferase MbtK) and Rv2416c (enhanced intracellular survival protein). It was previously shown that Rv1347c is involved in the mycobactin biosynthesis [54]. Seven acetyltransferases also belong to the category of essential genes according to the bibliometric approach [55]. Correspondence of acetyltransferases with virulence factors and essential genes are shown in Figure 4A and Supplementary Table S1. Essential genes are indispensable for survival. These essential genes are: *rv1653* (arginine biosynthesis bifunctional protein ArgJ), *rv2215* (dihydrolipoamide acyltransferase, DlaT), *rv3341* (homoserine O-acetyltransferase), *rv3546* (acetyl-CoA acetyltransferase), *rv1018c* (bifunctional protein GlmU), *rv2747* (amino-acid acetyltransferase), and *rv1347c*. Apart from Rv3546, other acetyltransferases are essential genes validated through high density mutagenesis experiment [56].

### Overlap of acetyltransferases with succinylation, acetylation, and glutarylation proteins

PTM (protein post-translational modification) functions in the regulation of different cellular processes in bacteria and eukaryotes [57]. Recently, our group identified 658 acetylated proteins with 1128 acetylation sites, 626 succinylated proteins with 1545 succinylation sites, and 24 glutarylated proteins with 41 glutarylation sites by using proteomics methods. To investigate whether three PTMs including acetylation, succinylation and glutarylation occur in these acetyaltransferases, we compared acetyltransferases with our previous data. The comparison showed that only one acetyltrasferase, Rv2215, can be acetylated, succinylated and glutarylated (Figure 4B and Supplementary Table S5). Rv2215 is dihydrolipoyllysine-residue acetyltransferase component of pyruvate dehydrogenase complex *M. tuberculosis* H37Rv *rv2215* mutant showed significant growth defect *in vitro* and more sensitive to nitrosative stress but not heat or INH [58]. In addition, *rv2215* mutant survival in bone marrow-derived macrophages from C57BL/6 and iNOS -/- mice was decreased [58]. Previous studies had demonstrated that the activities of metabolic enzymes such as acetyl-CoA synthetase (ACS) in *M. tuberculosis* can be regulated by PTMs [59,60]. Therefore, we guess that the enzyme activity of Rv2215 may be regulated by these PTMs. it is very necessary to study the role of these three PTMs in the regulation of Rv2215 by site-specific mutagenesis experiments *in vitro* and *in* vivo.

### Genomic context

To explore the occurrence and genome organization of these acetyltransferases, we performed analysis in the TB database (http://www.tbdb.org/) website. The results showed that the adjacent genes (at least one) of most acetyltransferases exist in other mycobacteria, even Rhodococcus and Streptomyces. For example, the adjacent genes of Rv1653c including Rv1652, Rv1654, Rv1655, and Rv1656 are highly conserved in *M. leprae* TN, *M. smegmatis* str. mc^2^155, *M. avium* 104, *Rhodococcus* sp.RHA1, and *Streptomyces avermitilis* MA-4680 (Supplementary Figure S2). However, the adjacent genes of several acetyltransferases are not conserved. For instance, there are no homologous proteins of the adjacent genes for Rv2416c.

### Meta-analysis of published omics study

To determine whether these acetyltransferases function during infection in *M. tuberculosis*, available data from previous transcriptomic and proteomic studies were used to analyze those acetyltransferases that show differential expression in infection models or under in *vitro* conditions mimicing aspects of infection. The analysis results are shown in Supplementary Table S2. Eighteen acetyltransferases showed differential expression in at least one of the experimental conditions while five acetyltransferases were essential for infection in mice model.

Seven acetyltransferases (Rv3523, Rv3546, Rv3556c, Rv1347c, Rv2416c, Rv1505c, and Rv3535c) are induced for infection in the BM macrophages model. In 2000, Wei et al. selected the *M. smegmatis* transformants containing an *M. tuberculosis* H37Rv plasmid library in human histocytic macrophage-like U-937 cell line, and found an enhanced intracellular survival gene (Eis), Rv2416c [61]. Both Eis enzymes from *M. tuberculosis* and *M. smegmatis* acetylated capreomycin and several lysine-containing compounds, and this acetylation of capreomycin was found at the ɛ-amine of the β-lysine side chain [61]. Recently, Sowajassatakul et al. found that overexpression of *eis* could occur without a mutation in the promoter region and be detectable in amikacin- and kanamycin-resistant *M. tuberculosis* clinical strain [62]. Ye et al. demonstrated that Eis proteins have the ability to acetylate many arylalkylamines, and are a novel family of arylalkylamine N-acetyltransferase AANAT (EC 2.3.1.87) [63]. Eis_Mtb from *M. tuberculosis* prefers to acetylate histamine and octopamine, while Eis_Msm from *M. smegmatis* uses tyramine and octopamine as substrates [63]. In addition, *M. tuberculosis* Eis can modulate autophagy, inflammation, and cell death through redox-dependent signal pathway to repress host innate immune defenses [64]. Kim et al. showed that *M. tuberculosis* Eis can acetylate DUSP16 (dual-specificity protein phosphatase 16)/MKP-7 (mitogen-activated protein kinase phosphatase-7) at Lys55, to inhibit JNK-dependent autophagy, phagosome maturation, and ROS (reactive oxygen species) generation. It will be very interesting to study the physiological role of the other essential acetyltransferases.

## Conclusion

By *in silico* methods, acetyltransferases were predicted from the available complete proteome of *M. tuberculosis*. This is the first study establishing whole map of *M. tuberculosis* acetyltransferases and their evolution, antigen, and genomic context. We have identified 47 acetyltransferases in *M. tuberculosis*. Several acetyltransferases have well established roles in virulence, antibiotic resistance, and metabolism.

Aminoglycoside acetyltransferases, members of the GNAT superfamily, can confer resistance to aminoglycoside antibiotics in bacteria [65]. CysE (Serine acetyltransferase), one enzyme involved in L-cysteine biosynthesis pathway, exists in bacteria and plants, but not in humans [66]. CysE can catalyze the chemical reaction transferring the acetyl from AcCoA (acetyl-CoA) to L-Ser (L-serine). Rv2335 was identified and characterized as an serine acetyltransferase in *M. tuberculosis* [67], and *M. smegmatis* homologous *cysE* knockout mutant strain showed significant morphological changes and suppressed the bacteria growth [68]. NCBI blast results showed that homologous proteins of these acetyltransferases are not found in human and other mammals. This implies that those acetyltransferases can become the potential drug targets.

In the present study, we found that Rv2215 can be acetylated, succinylated, and glutarylated. It have been demonstrated that lysine acetylation in protein catalyzed by specific acetyltransferases play an important role in bacterial metabolism, stress response, and virulence [69,70]. Rv0998, one GNAT family member having a cyclic nucleotide binding domain, can acetylate several mycobacterial fatty acyl CoA ligases (FACLs), thereby repressing FACLs enzyme activities and regulating fatty acid metabolism [71]. Therefore, it will be very interesting to study whether Rv0998 is the key enzyme responsible for acetylating DlaT protein. In 2015, Tran et al. using high-throughput screen method identified two compounds (6624116, 5655606) that can inhibit the acetyltransferase activity of GlmU in *M. tuberculosis* and increase the anti-TB activity combination with other anti-TB drugs [72]. In 2016, Garzan et al. discovered two Eis inhibitors as kanamycin adjuvants to kill drug-resistant *M. tuberculosis* through high-throughput screening [73]. Later, they developed pyrrolo (1,5-a) pyrazine-based analogues as new effective inhibitors of Eis [74]. These inhibitors may become promising anti-TB drugs. However, some of these acetyltransferases were classified into hypothetical proteins without any predicted function. Therefore, it will be interesting to further study the functional role of these acetyltransferases in the adaptation of the *M. tuberculosis* in *in vivo* systems.

## Supplementary Material

Supplementary Figures S1-S2

Supplementary Tables S1-S5

## Abbreviations

AcCoA: acetyl-CoA
AG: arabinogalactan
CysE: serine acetyltransferase
DUSP16: dual-specificity protein phosphatase 16
Eis: enhanced intracellular survival gene
FACLs: fatty acyl CoA ligases
GlmU: N-acetylglucosamine-1-phosphate uridyltransferase
GNAT: GCN5-related acetyltransferase
MKP-7: mitogen-activated protein kinase phosphatase-7
PG: peptidoglycan
PTM: protein post-translational modification
PTMs: post-translational modifications
TB: tuberculosis
