# Supplementary figures and images for: Comprehensive analysis of protein acetyltransferases of human pathogen Mycobacterium tuberculosis
Source: Biosci Rep. 2019 Dec 20;39(12):BSR20191661. doi: 10.1042/BSR20191661 (PMC6923341; doi:10.1042/BSR20191661)

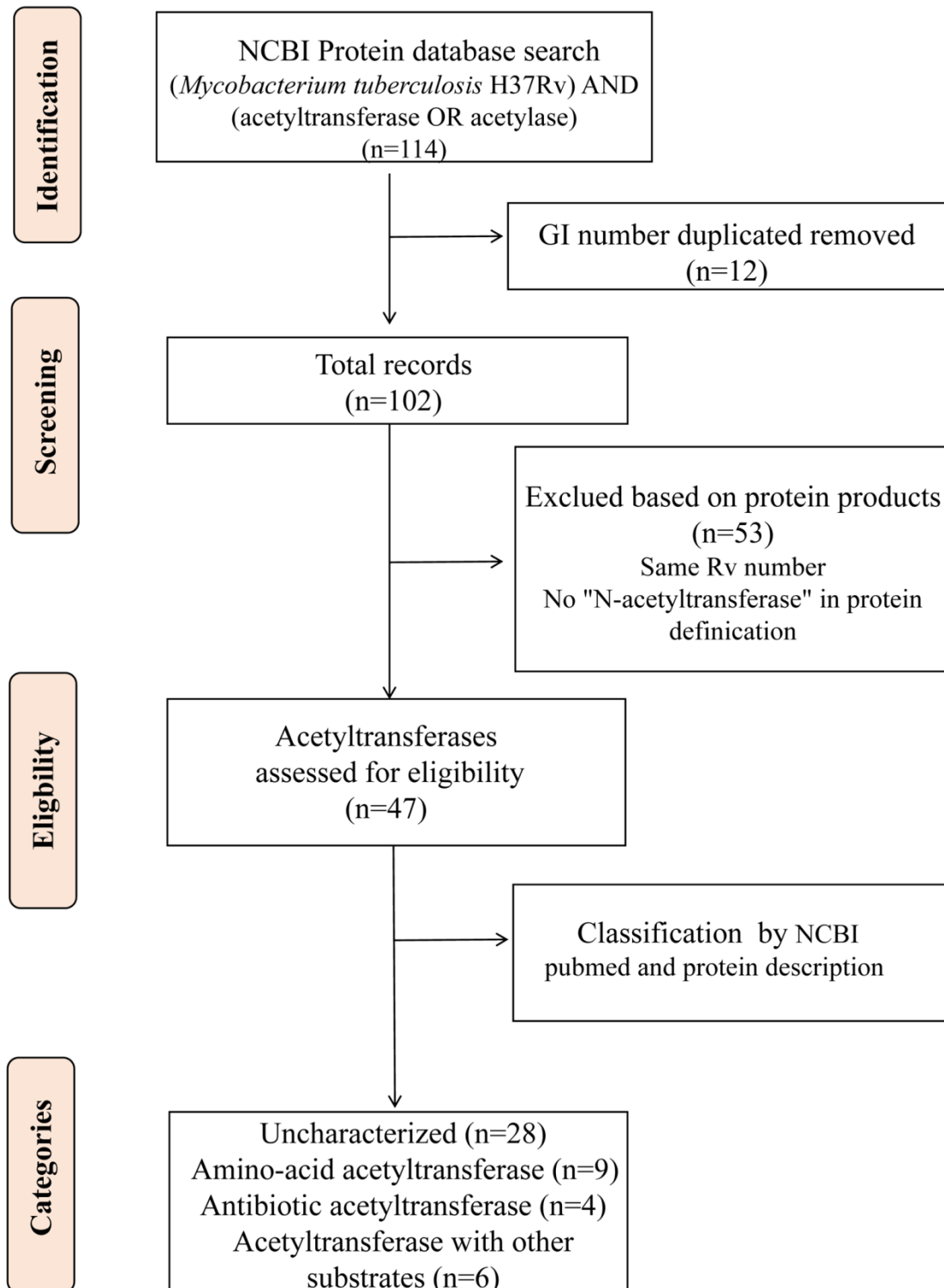

Rv1653c

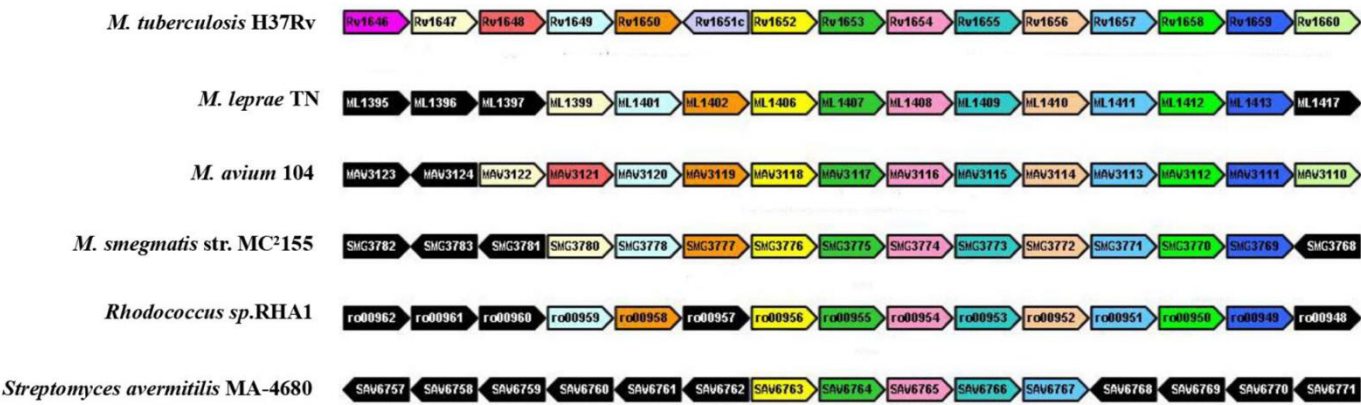

Supplement: Supplementary Figures S1-S2 [file BSR-2019-1661_supp.pdf]
