# Supplementary material for: Comprehensive analysis of protein acetyltransferases of human pathogen Mycobacterium tuberculosis
Source: Biosci Rep. 2019 Dec 20;39(12):BSR20191661. doi: 10.1042/BSR20191661 (PMC6923341; doi:10.1042/BSR20191661)
Supplement: Supplementary Tables S1-S5 [file BSR-2019-1661_supp1.zip › BSR-2019-1661_suppST5.doc]

Table S5. The detailed protein posttranslational modification sites in Rv2215.

| Gene name | Modified sequence | Position | PTMs |
| --- | --- | --- | --- |
| Rv2215 | AK(kg)AAFAER | 361 | Glutarylation |
| Rv2215 | PAPEPPPVQPTSGAPAGGDAK(su)PVLMPELGESVTEGTVIR | 122 | Succinylation |
| Rv2215 | AK(ac)AAFAER  FLTTIK(ac)HR | 361  539 | Acetylation |
